# Psychosocial functioning of individuals at risk of developing compulsive buying disorder

**DOI:** 10.1192/j.eurpsy.2025.983

**Published:** 2025-08-26

**Authors:** K. Rachubińska, A. M. Cybulska, D. Schneider-Matyka, M. Stanisławska, E. Grochans

**Affiliations:** 1 Department of Nursing, Pomeranian Medical University, Szczecin, Poland

## Abstract

**Introduction:**

Personality-related correlates are significant factors associated with compulsive buying. The Big Five personality traits can be a risk factor or a protective factor for addiction.

**Objectives:**

This study aimed to establish the connection between depressiveness, workaholism, eating disorders, and personality traits, according to the five-point model called the Big Five, in women with a risk of compulsive buying disorder.

**Methods:**

The study was conducted on 556 Polish women from the West Pomeranian Voivodeship. The study employed the diagnostic survey method using a questionnaire technique including Personality Inventory NEO-FFI, the Buying Behaviour Scale, the Beck Depression Inventory I-II, the Three-Factor Eating Questionnaire, and a self-questionnaire.

**Results:**

The analysis revealed the risk of compulsive buying being accompanied by a higher median score for depressiveness, neuroticism, Cognitive Restraint of Eating, Uncontrolled Eating, and a risk of workaholism. A lower score in the respondents in the compulsive buying risk group was observed in an assessment of agreeableness and conscientiousness. Work addiction was exhibited by 26% of people with compulsive buying disorder vs. 12% of people without it.

**Table 1.** Descriptive statistics for selected scales with respect to the risk of the compulsive buying disorder.

**Table:** 

Selected Scales	Total (n = 556)	Norm (n = 483) Group 1	Risk of Compulsive Buying (n = 73) Group 2	*p*
BDI I-II Me (Q1–Q3)	4.5 (1.0–10.0)	4.0 (1.0–9.0)	8.0 (1.0–15.0)	0.021
Neuroticism acc. to NEO-FFI, Me (Q1–Q3)	21.0 (15.0–28.0)	21.0 (15.0–28.0)	24.0 (20.0–32.0)	0.003
Openness to experience acc. to NEO-FFI, Me (Q1–Q3)	26.0 (23.0–31.0)	26.0 (23.0 -31.0)	26.0 (23.0–30.0)	0.774
Agreeableness acc. to NEO-FFI, Me (Q1–Q3)	30.0 (27.0–34.0)	31.0 (27.0 -34.0)	27.0 (24.0–32.0)	<0.001
Conscientiousness acc. to NEO-FFI, Me (Q1–Q3)	34.0 (29.0–38.0)	34 (30.0–39.0)	30.0 (25.0–38.0)	0.028
Cognitive Restraint of Eating acc. to TFEQ-13, Me (Q1–Q3)	6.0 (4.0–8.0)	6.0 (4.0–8.0)	7.0 (5.0 -9.0)	0.004
Uncontrolled Eating acc. to TFEQ-13, Me (Q1–Q3)	5.5 (4.0–7.0)	5.0 (4.0–7.0)	7.0 (5.0 -8.0)	0.019
WART, Me (Q1–Q3)	53.0 (45.0–62.0)	51.0 (44.0–61.0)	60.0 (51.0–66.0)	<0.001
Addiction to work acc. to WART, n (%)	79	60 (12.42%)	19 (26.03%)	<0.001

BDI I-II—Beck Depression Inventory, NEO-FFI—Personality Inventory NEO-FFI, TFEQ-13—Three-Factor Eating Questionnaire, WART—Work Addiction Risk Test, Me—median, Q1—quartile first, Q3—quartile third, n—number of patients, *p*—statistic

**Conclusions:**

This study found that a high risk of compulsive buying disorder is accompanied by a high risk of moderate depressiveness, neuroticism, Cognitive Restraint of Eating, Uncontrolled Eating, and workaholism. It also confirmed the view that compulsive buying is a behavioural addiction which is a consequence of ineffective coping and being dissatisfied with one’s social life.

**Disclosure of Interest:**

None Declared

